# HPLC Method for the Simultaneous Determination of Ten Annonaceous Acetogenins after Supercritical Fluid CO_2_ Extraction

**Published:** 2010-09

**Authors:** Haijun Yang, Ning Zhang, Qingqi Zeng, Qiping Yu, Shihuai Ke, Xiang Li

**Affiliations:** 1 *Department of Pharmacy, College of Jiangsu Jiankang Profession, Nanjing, P. R. China;*; 2 *Department of Pharmacy, NanJing University of Chinese Medicine, Nanjing, P. R. China*

**Keywords:** *Annonaceae plant* seeds, supercritical fluid CO_2_ extraction, quantification, HPLC–DAD, annonaceous acetogenins

## Abstract

Annonaceous acetogenins (ACGs) isolated from Annonaceae plants exhibited a broad range of biological bioactivities such as cytotoxic, antitumoral, antiparasitic, pesticidal and immunosuppresive activities. However, their structures were liable to change at more than 60°C and their extraction yields were low using traditional organic solvent extraction. In the present study, all samples from *Annona* genus plant seeds were extracted by supercritical carbon dioxide under optimized conditions and a high-performance liquid chromatography (HPLC) method was established for simultaneously determining ten ACGs. All of the ten compounds were simultaneously separated on reversed-phase C_18_ column (250 mm × 4.6 mm, 5 μm) with the column temperature at 30°C. The mobile phase was composed of (A) methanol and (B) distilled water, the flow rate was 1.0 ml/min and the detection wavelength was set at 220 nm. All calibration curves showed good linear regression (γ>0.9995) within the test range. The established method showed good precision and accuracy with overall intra-day and inter-day variations of 0.99-2.56% and 1.93-3.65%, respectively, and overall recoveries of 95.16-105.01% for the ten compounds analyzed. The established method can be applied to evaluate the intrinsic quality of *Annonaceae* plant seeds. The determination results recover the content-variation regularities of various ACGs in different species, which are helpful to choose the good-quality *Annonaceae* plant seeds for anticancer lead compound discovery.

## INTRODUCTION

Annonaceous acetogenins (ACGs) constitute a series of natural products isolated exclusively from *Annonaceae* plants (1-4), which are comprised of some 130 genera and include over 2300 species and are widely distributed in tropical and sub-tropical regions. More than 500 ACGs have been isolated and identified from this plant family, mostly from the seeds and stem bark. Chemically, the annonaceous acetogenins are white, waxy, derivatives of long-chain (C35 or C37) fatty acids. They are usually characterized by a long aliphatic chain bearing a terminal methyl-substituted α, β - unsaturated γ - lactone ring with one, two, three tetrahydrofuran (THF) or tetrahydropyran (THP) rings. ACGs exhibited a broad range of biological activities such as cytotoxic, antitumoral, antiparasitic, pesticidal and immunosuppresive activities (5-10). Especially, their ability to inhibit multiple drug resistant (MDR) tumor cell lines (11, 12) has attracted much attention of chemists and biologists. The ACGs are the most powerful known inhibitors of complex I (NADH: biquinone oxidoreductase) in mammalian and insect mitochondrial electron transport system (13-15). In addition, they are potential inhibitors of NADH oxidase of the plasma membranes of cancer cells, the inhibition results in a depletion of ATP levels which causes arrest in the cell cycle at the G1 phase, and subsequently apoptosis is induced (16-20). So, ACGs are regarded as a likely source for the development of potential drugs.

**Figure 1:**
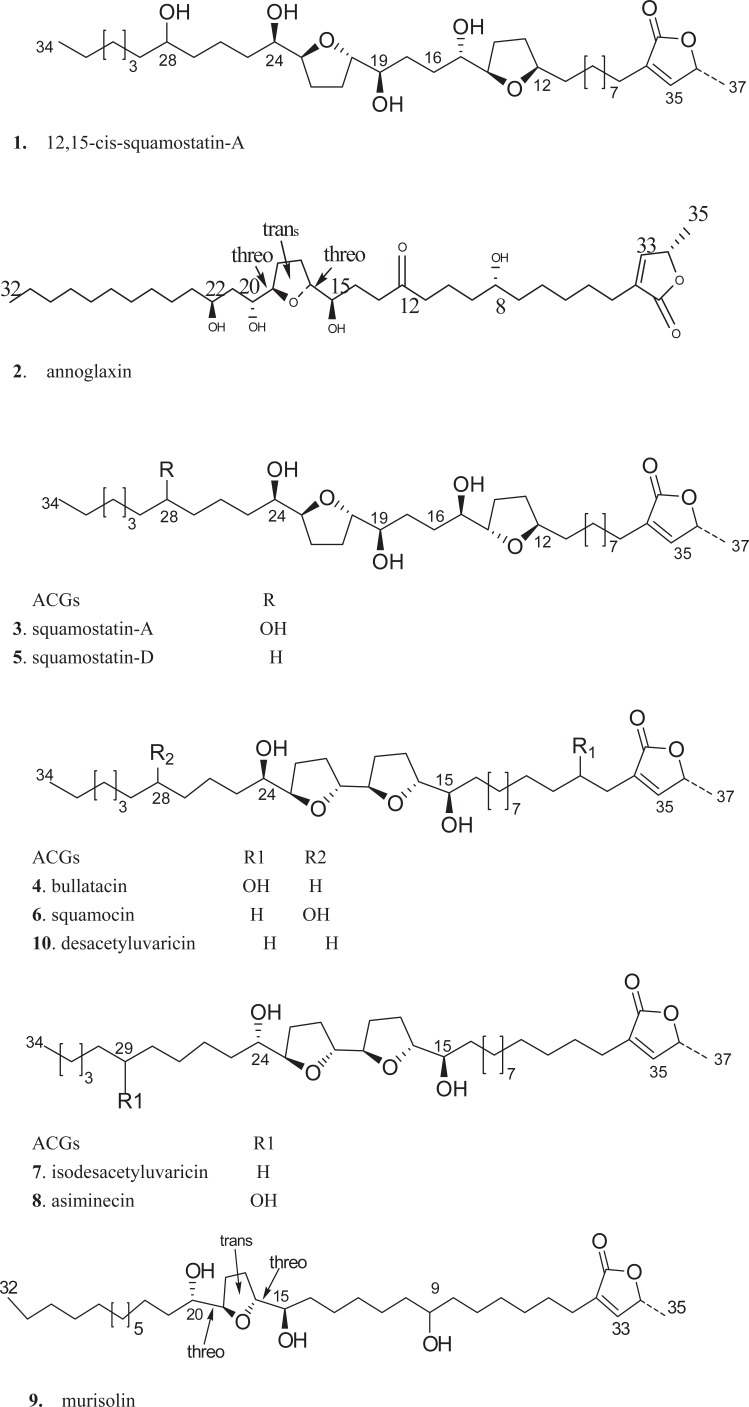
Chemical structures of annonaceous acetogenins (ACGs) from Annona squamosa seeds.

However, ACGs are low polarity compounds and their structures were liable to change at more than 60°C and their extraction yields were low using classical organic solvent extraction method. As an alternative of traditional extraction method, supercritical fluid CO_2_ extraction (SFE), an extraction technique under low temperature, has recently used in the extraction of bioactive constituents from herbal medicines for its small amount of solvent consumption, automated sample handling and high extractive efficiency. So in the present study all samples were extracted by SFE and the optimal technology was concluded by our previous research (21). To our knowledge, only a few analytical methods are available for determination ACGs, previously reported analytical methods were just developed to determine the total ACGs by spectrophoto- metric analysis (22), which was instable and to quantify just three types of ACGs by HPLC method (20). So in order to assess the intrinsic quality of different *Annonaceae* plant seeds and meet the regulatory requirements for investigating *Annonaceae* plant seeds, a HPLC method was developed for simultaneous determination ten major ACGs in *Annona* genus plant seeds distributed in south of China. Figure 1 illustrates the representatives of the ten ACGs which represent the main structural types of bio- active ACGs and their contents are considerable in *Annonaceae* plant seeds.

## EXPERIMENTAL

### Chemicals and reagents

The ten reference compounds (12,15-*cis*-squamostatin-A, annoglaxin, squamostatin-A, bullatacin, squamocin, squamostatin-D, isodesacetyluvaricin, asiminecin, murisolin and desacetyluvaricin) were isolated from *Annon squamosa* seeds by our laboratory and their structures were established based on spectroscopic analysis, the purity of each reference compound was determined to be above 99% by HPLC analysis and confirmed by LC-MS, NMR spectroscopy. HPLC-grade methanol was purchased from Hanbang Science and Technology Company (Nanjing, China), the deionized water was purified using a Milli-Q Plus 185 system from Millipore (Milford, MA, USA).

### Plant material

Five *Annona* genus plant (*Annona squamosa, A. muricata, A. glabra, A. reticulata and A. bullata*) seeds were collected from Jiangsu, Hainan, Guangdong and Yunnan provinces, P. R. China and were identified by Professor Jianwei Chen (NanJing university of Chinese Medicine, Nanjing, China). After collection, the seeds were allowed to dry at ambient temperature for about one week and were then crushed and immediately extracted.

### Apparatus and Chromatographic conditions

A supercritical fluid extractor SFE-2 (Applied Separation, USA) which is capable of pressure up to 680 bar and temperature up to 240°C, static and dynamic extraction with flow from 0 to 10 L/min (gaseous carbon dioxide) and extraction vessels from 5 ml to 1l were used. An Agilent 1200 liquid chromatograph system (Agilent technologies, CA, USA) consisting of binary pump, an auto-sampler and diode-array detector was used. The column configuration consisted of an Agilent Zorbax Extend reversed-phase C_18_ column (250 mm × 4.6 mm, 5 μm). Detection wavelength was set at 220 nm. The mobile phase consisted of A (methanol) and B (deionized water), using a linear gradient: 0-40 min (85% A), 40-60 min (85% A-95% A). The flow rate was 1.0 ml/min. The column temperature was maintained at 30°C.

### Preparation of standard solutions

A mixed standard stock solution containing 12,15-*cis*-squamostatin-A (a), annoglaxin (b), squamostatin-A (c), bullatacin (d), squamostatin-D (e), squamocin (f), isodesacetyluvaricin (g), asiminecin (h), murisolin (i) and desacetyluvaricin (j) was prepared in methanol. Working standard solutions were prepared by diluting the mixed standard solution with methanol to give six different concentrations within the ranges: a, 3.8-45.6 μg/ml; b, 2.1-26.7 μg/ml; c, 3.2-36.4 μg/ml; d, 4.5-51.7 μg/ml; e, 2.7-29.2 μg/ml; f, 0.58-17.3 μg/ml; g, 2.5-18.9 μg/ml; h, 4.3-48.9 μg/ml; i, 3.3-41.8 μg/ml and j, 3.5-44.6 μg/ml for calibration curves. The standard solutions were filtered through a 0.45 μm membrane prior to injection. The standard stock and working solutions were stored at 4°C.

### Preparation of sample solutions

The dried powder of *Annona* plant seeds (100 g, 20 mesh) was accurately weighed and extracted by SFE under optimized conditions (extraction pressure: 30 Mpa; extraction temperature: 35°C; extraction time: one hour; 20 ml 95% ethanol modifier) (21). After evaporating ethanol to dryness by a rotary evaporator, residue was dissolved in methanol in a 25 ml flask, and then filtrated through a 0.45 micro-m millipore filter before HPLC injection. Three aliquots of the solution (20 μl) were injected to RP-HPLC-DAD system.

## RESULTS AND DISCUSSION

### Optimization of separation condition

**Figure 2:**
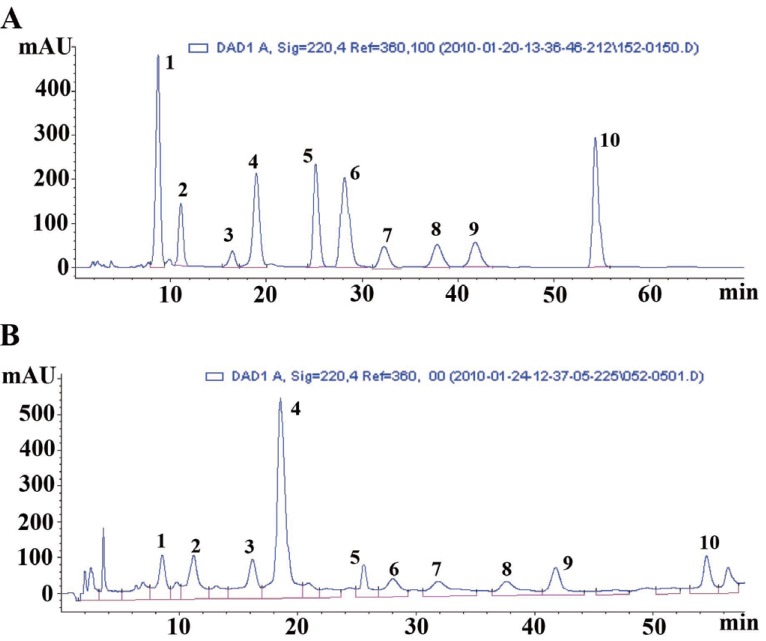
Reprehensive HPLC chromatograms of reference compounds (A) and SFE extract of Annona squamosa seeds (B). Peak identification: (1) 12,15-cis-squamostatin-A; (2) annoglaxin (3) squamostatin-A; (4) bullatacin; (5) squamostatin-D; (6) squamocin; (7) isodesacetyluvaricin; (8) asiminecin; (9) murisolin and (10) desacetyluvaricin.

Different mobile phase compositions were examined: methanol-water and acetonitrile-water. As a result, the 85% methanol and 15% water system could give best separation of the ten reference compounds in 60 min. Furthermore, other chromatographic variables were also optimized, including analytical columns (Hanbon Hedera ODS-2, Hanbon Lichrospher C_18_ and Agilent Zorbax Extend C_18_), the colum temperatures (20°C, 25°C and 30°C) and the flow rates (0.8 ml/min and 1.0 ml/min). Eventually, the optimal separation was achieved on an Agilent Zorbax Extend C_18_ colum (250 mm × 4.6 mm, 5 μm) at a column temperature of 30°C with a flow rate of 1.0 ml/min. According to the absorption maxima of ten reference compounds on the UV spectra with three-dimensional chromatograms of HPLC-DAD detection, the wavelength was set at 220 nm. Representative chromatograms for the standard analytes and for a sample were shown in Fig. 2. Fig. 2A displayed that the ten standard analytes were well separated and the resolution between any two compounds was greater than 1.5. Other compounds in the sample did not interfere with analysis of the ten standard analytes, as shown in Fig. 2B. The chromatographic peaks were identified by comparing their retention time with that of each reference compound, which was eluted in parallel with the optimised mobile phases. In addition, spiking samples with the reference compounds showed no additional peaks, which further confirmed the identities of the analytes’ peaks.

### Calibration curves, limits of detection and quantification

The calibration curves were performed with 6 different concentrations in triplicate. All calibration curves were obtained from peak areas (Y) of the standard solutions versus the concentrations for reference compounds. Linear regression analysis for each of the ten reference compounds was performed by the external standard method. The limit of detection (LOD) was determined at a signal-to-noise ratio of 3 and the limit of quantificaion (LOQ) was determined as the lowest concentration in the linear range of each analyte. The calculated results are given in Table 1. All ten reference compounds showed good linearity (r>0.9995) in a relatively wide concentration range. The limits of detection (LOD) and quantification (LOQ) of the ten analytes were 0.11-0.42 μg/ml and 0.58-4.5 μg/ml, respectively. The LOD and LOQ were reported in Table 1.

**Table 1:** Calibration curves of ten analytes

Analytes	Calibration curves	r	Linearrange (μg mL^-1^)	LOD (μg mL^-1^)	LOQ (μg mL^-1^)

1. 12,15-cis-squamostatin-A	Y=6365.9x-777.99	0.9990	3.8-45.6	0.11	3.8
2. annoglaxin	Y= 3031.1x-59.21	0.9990	2.1-26.7	0.15	2.1
3. squamostatin-A	Y= 586.9x-17.26	0.9990	3.2-36.4	0.42	3.2
4. bullatacin	Y=3645.3x-428.66	0.9995	4.5-51.3	0.19	4.5
5. squamostatin-D	Y=4683.5x + 37.20	0.9995	2.7-29.2	0.33	2.7
6. squamocin	Y=698.5x-33.82	0.9999	0.58-17.3	0.19	0.58
7. isodesacetyluvaricin	Y=5002.3x-289.36	0.9999	2.5-18.9	0.23	2.5
8. asiminecin	Y=482.5x + 711.32	0.9999	4.3-48.9	0.27	4.3
9. murisolin	Y=1276.3x + 68.51	0.9999	3.3-41.8	0.21	3.3
10. desacetyluvaricin	Y=3842.0x-155.74	0.9999	3.5-44.6	0.26	3.5

Y is peak area; x is concentration of the analytes (μg/ml); r is the correlation coefficient of the equation; LOD refers to limit of detection and LOQ refers to limit of quantification.

### Precision and stability

Intra-day and inter-day variations were chosen to determine the precision of the developed method by analyzing certain concentrations of standard solution. For intra-day variation, the standard solution was analyzed for six times within one day, the inter-day variation was determined in three consecutive days. The overall relative standard deviations of the intra-day and inter-day were less than 3.66%. The results were shown in Table 2.

**Table 2:** Precision and recovery data of the ten analytes in *Annonaceae Squamosa* seeds

Analytes	Precision		Recovery	
	Intra-day (n=6) R.S.D. (%)	Inter-day (n=3) R.S.D. (%)	Mean (%)	R.S.D. (%)

1. 12,15-cis-squamostatin-A	1.45	2.41	95.2	4.2
2. annoglaxin	1.21	2.91	96.1	3.1
3. squamostatin-A	1.56	1.97	99.3	3.8
4. bullatacin	1.74	3.38	96.9	3.0
5. squamostatin-D	1.26	2.41	103.1	2.5
6. squamocin	1.70	1.93	105.1	2.7
7. isodes-acetyluvaricin	1.12	2.21	99.7	3.1
8. asiminecin	2.56	3.21	98.4	2.1
9. murisolin	1.77	3.65	95.4	3.7
10. desacetyluvaricin	0.99	2.13	104.9	2.7

Stability study was performed with sample solution at room temperature and analyzed at 0 h, 2 h, 4 h, 8 h, 12 h, 24 h and 48 h within two days, respectively. Variations were expressed by relative standard deviations (R.S.D.). The R.S.D. of stability was not more than 4.21% for all analytes.

### Recovery

An appropriate amount of sample was weighed and spiked with known amount of each standard compound. They were then treated and analyzed as described above. Each sample was analyzed in triplicate. The average recoveries were estimated by the formula: recovery (%)=(amount found - original amount)/amount spiked × 100%. The total amount of each analyte was calculated from the corresponding calibration curve. The overall recoveries lay between 95.2% and 105.1% for all reference compounds, with R.S.D. less than 4.5% indicating that the established method was accurate enough for the determination of the ten annonaceous acetogenins in *Annona* plant seeds. The results of recovery test were shown in Table 2.

### Sample analysis

The established method has been successfully applied to the simultaneous determination of ten annonaceous acetogenins from five different *Annona* plant seeds. The contents (n=3) of ten annonaceous acetogenins were listed in Table 3. It can be seen that all ten compounds could be detected in all samples. The contents of these components varied in different *Annona* plant seeds. From the results, it was easy to note that bullatacin (3) was the most dominant compound in all samples. Its content ranged from 0.33 to 0.57 mg/g. Besides, the total content of the ten compounds ranged from 2.04 to 2.85 mg/g which might be due to the differences in soils and climates in each region. Especially, the total content of the ten compounds in the *Annona squamosa* seeds was higher than other four *Annona* plant seeds. Thus it is necessary to control the main bioactive ACGs in different *Annonaceae* plant seeds by good agricultural practice (GAP). Then the quality of *Annonaceae* plant seeds could be assured.

**Table 3:** Contents of ten analytes in samples of *Annonaceae* seeds (mg/g)

Sample	origin	1	2	3	4	5	6	7	8	9	10	total

A.squamosa	Guangdong	0.22	0.30	0.25	0.57	0.20	0.37	0.22	0.17	0.27	0.28	2.85
A. glabra	Hainan	0.17	0.18	0.13	0.46	0.13	0.26	0.20	0.24	0.15	0.27	2.18
A. muricata	Hainan	0.21	0.21	0.10	0.37	0.17	0.27	0.19	0.20	0.19	0.18	2.09
A.reticulata	Yunnan	0.27	0.28	0.12	0.33	0.11	0.31	0.14	0.28	0.15	0.14	2.13
A.bullata	Jiangsu	0.16	0.23	0.14	0.40	0.08	0.33	0.13	0.18	0.11	0.27	2.04

## CONCLUSIONS

A validated analytical method for qualification and quantification of annonaceous acetogenins from different *Annona* plant seeds has been developed, the new method was evaluated to be precise and accurate and successfully applied to determine the contents of ten major ACGs from five different *Annona* plant seeds. It was a convenient and precise method to assess the quality of different *Annonaceae* plant seeds.

## References

[1] Zafra-Polo MC, Gonz’alez MC, Estornell E, Sahpaz S (1996). Acetogenins from Annonaceae, Inhibitors of Mitochondrial Complex I. Phytochemistry.

[2] Zafra-Pola MC, Figad`ere B, Gallardo T, Tormo JR (1998). Natural Acetogenins from Annonaceae, synthesis and mechanisms of action. Phytochemistry.

[3] Zeng L, Ye Q, Oberlies NH, Shi G, Gu ZM, He K (1996). McLaughlin, Recent Advances in Annonaceous Acetogenins. Nat. Prod. Rep.

[4] Alali FQ, Liu XX, McLaughlin JL (1999). Annonaceous Acetogenins: Recent Progress. J. Nat. Prod.

[5] Tormo JR, Gallardo T, Gonz’alez MC, Bermejo A (1999). Annonaceous acetogenins as inhibitors of mitochondrial complex I. Curr. Top. Phytochem.

[6] Londerhausen M, Leicht W, Lieb F, Moeschler H (1991). Molecular mode of action of annonins. Pestic. Sci.

[7] Degli Esposti M, Ghelli A, Ratta M, Cortes D (1994). Natural substances (acetogenins) from the family Annonaceae are powerful inhibitors of mitochondrial NADH dehydrogenase (Complex I). Biochem. J.

[8] Leonard JV, Schapira AHV (2000). Mitochondrial respiratory chain disorders I: mitochondrial DNA defects. Lancet.

[9] Lenaz G, Fato R, Barraca A, Genova ML (2004). Mitochondrial quinone reductases: Complex I. Methods Enzymol.

[10] Brandt U, Kerscher S, Zwicker K (2003). Proton pumping by NADH: ubiquinone oxidoreductase. A redox driven conformational change mechanism. FEBS Lett.

[11] Estornell E (2000). Mitochondrial. Complex I: Newinsights frominhibitor assays. Protoplasma.

[12] Tormo JR, Estornell E (2000). New evidence for the multiplicity of ubiquinone- and inhibitor-binding sites in the mitochondrial complex I. Arch. Biochem. Biophys.

[13] Yagi T, Matsuno-Yagi A (2003). The proton translocating NADHquinone oxidoreductase in the respiratory chain: The secret unlocked. Biochemistry.

[14] Schuler F, Casida JE (2001). The insecticide target in the PSST subunit of complex I. Pest Manage. Sci.

[15] ino T, Nishioka T, Miyoshi H (2003). Characterization of inhibitor binding sites ofmitochondrial complex I using fluorescent inhibitor. Biochim. Biophys. Acta.

[16] Ogiso EN, Sakamoto K, Matsuno-Yagi A, Miyoshi H (2003). The ND5 subunit was labeled by a photoaffinity analogue of fenpyroximate in bovine mitochondrial Complex I. Biochemistry.

[17] Tormo JR, Gallardo T, Arag’on R, Cortes D (1999). Specific interactions of monotetrahy -drofuranic Annonaceous acetogenins as inhibitors of mitochondrial complex I. Chem. Biol. Interact.

[18] Tormo JR, Estornell E, Gallardo T, Gonz’alez MC (2001). Lactone- functionalized antitumoral acetogenins are the most potent inhibitors of mitochondrial complex I. Bioorg.Med. Chem. Lett.

[19] Tormo JR, Gonz’alez MC, Cortes D, Estornell E (1999). Kinetic characterization of mitochondrial Complex I inhibitors using Annonaceous acetogenins. Arch. Biochem. Biophys.

[20] Tormo JR, Zafra-Polo MC, Serrano A, Estornell E (2000). Epoxy-acetogenins and other polyketide epoxy-derivatives as inhibitors of the mitochondrial respiratory chain complex I. Planta Med.

[21] Yang HJ, Li X (2008). Study the optimum extraction of annonaceous acetogenins from seeds of Annona squamosa L by Supercritical Fluid CO2 extraction (SFE). J. US-China Sci. Med.

[22] Sun L, Yu JG, Li DY, Li J (2001). Determination of annonaceous acetogenins in annonnace plant by HPLC. Acta Pharm. Sin.

